# Model Sensitivity and Use of the Comparative Finite Element Method in Mammalian Jaw Mechanics: Mandible Performance in the Gray Wolf

**DOI:** 10.1371/journal.pone.0019171

**Published:** 2011-04-29

**Authors:** Zhijie Jack Tseng, Jill L. Mcnitt-Gray, Henryk Flashner, Xiaoming Wang, Reyes Enciso

**Affiliations:** 1 Integrative and Evolutionary Biology Program, Department of Biological Sciences, University of Southern California, Los Angeles, California, United States of America; 2 Department of Vertebrate Paleontology, Natural History Museum of Los Angeles County, Los Angeles, California, United States of America; 3 Departments of Kinesiology, Biological Sciences, and Biomedical Engineering, University of Southern California, Los Angeles, California, United States of America; 4 Department of Aerospace and Mechanical Engineering, University of Southern California, Los Angeles, California, United States of America; 5 Ostrow School of Dentistry, University of Southern California, Los Angeles, California, United States of America; Utah State University-College of Eastern Utah, United States of America

## Abstract

Finite Element Analysis (FEA) is a powerful tool gaining use in studies of biological form and function. This method is particularly conducive to studies of extinct and fossilized organisms, as models can be assigned properties that approximate living tissues. In disciplines where model validation is difficult or impossible, the choice of model parameters and their effects on the results become increasingly important, especially in comparing outputs to infer function. To evaluate the extent to which performance measures are affected by initial model input, we tested the sensitivity of bite force, strain energy, and stress to changes in seven parameters that are required in testing craniodental function with FEA. Simulations were performed on FE models of a Gray Wolf (*Canis lupus*) mandible. Results showed that unilateral bite force outputs are least affected by the relative ratios of the balancing and working muscles, but only ratios above 0.5 provided balancing-working side joint reaction force relationships that are consistent with experimental data. The constraints modeled at the bite point had the greatest effect on bite force output, but the most appropriate constraint may depend on the study question. Strain energy is least affected by variation in bite point constraint, but larger variations in strain energy values are observed in models with different number of tetrahedral elements, masticatory muscle ratios and muscle subgroups present, and number of material properties. These findings indicate that performance measures are differentially affected by variation in initial model parameters. In the absence of validated input values, FE models can nevertheless provide robust comparisons if these parameters are standardized within a given study to minimize variation that arise during the model-building process. Sensitivity tests incorporated into the study design not only aid in the interpretation of simulation results, but can also provide additional insights on form and function.

## Introduction

Finite element analysis (FEA), the discretization of structures and approximation of their mechanical behavior (the response of structure to load), has traditionally been an analytical technique in the engineering disciplines as an important component of the development process to improve design. More recently, however, its use in functional studies of biological structures has become more common [1]–[4]. FEA has been applied in vertebrate biomechanics research across diverse taxonomic groups, including crocodiles [5], non-avian dinosaurs [6]–[11], birds [12], lizards [13], [14], fishes [15], and a variety of mammals [16]–[26]. FEA complements *in vivo* experimental studies by allowing simulations using user-defined input assumptions regarding the study system, which could otherwise be impossible to implement. Currently, most studies of this type address the mechanical behavior of the craniodental system.

Given the diverse functional questions that could be examined using the FE approach, the current available data from FE publications are largely incomparable across studies precisely because of the comparative nature of current applications. Even within narrow clades of closely related genera and species, lack of absolute values from FE results means published stress and strain values cannot be used to evaluate relative performance of models across different studies (e.g. models of felid species in McHenry et al., 2007 versus those in Slater and Van Valkenburgh, 2009) [18], [22]. In many studies, different approaches in how muscles and constraints are modeled also make comparisons difficult. Furthermore, the diversity of taxonomic groups that can potentially be studied using this technique, accompanied by the different software programs and protocols used by researchers in FE model construction, further complicates any attempts at the synthesis of current FE knowledge across vertebrate groups. The current diversity of input assumptions in FE models used in comparative biology suggests a need to quantify the sensitivity of performance measures to those parameters, in order to build a general context for comparing results within and across different studies.

Several previous studies have addressed the choice of model parameters and their implications for comparing FE analytical results to those obtained from *in vivo* experiments for masticatory muscle forces [27], bite forces [28], and elastic bone properties [29]. However, few have addressed the comparison of relative values in the growing literature on vertebrate FE models, which is becoming more numerous given the flexibility of this approach in allowing tests of form and function [30]. In one attempt, Sellers and Crompton [31] conducted a sensitivity study of human bite force prediction with FEA using a large number of combinations of model parameters and found that masticatory muscle insertion points, as well as the modeled mobility of the temporomandibular joints (TMJ), had a large effect on resulting jaw forces. Even if current FEA applications in vertebrate functional morphology cannot provide accurate mechanical values for comparing to experimental results, and in most cases FE models do not have corresponding *in vivo* data for validation tests, standardized comparisons can nevertheless be highly informative [30]. Furthermore, a comparative approach has the advantage of being able to include extinct forms for which material properties and other parameters cannot be directly obtained.

**Figure 1:**
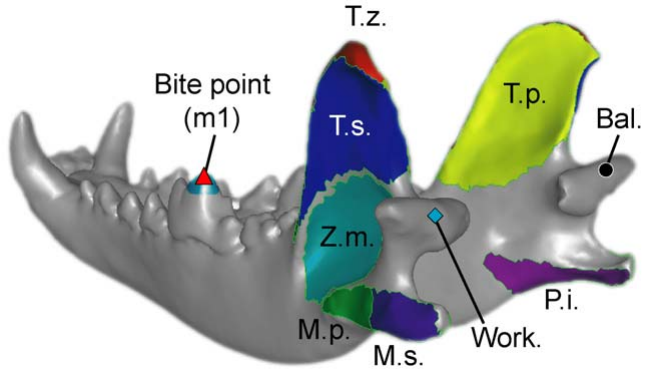
Mandible model used in the study. **Bal.**, balancing-side joint; **Work.**, working-side joint; **m1**, lower first molar (carnassial); **M.p.**, deep masseter; **M.s.**, superficial masseter; **P.i.**, internal pterygoid; **T.p.**, deep temporalis; **T.s.**, superficial temporalis; **T.z.**, zygomatic part of temporalis; **Z.m.**, zygomaticomandibularis. Temporalis and masseter muscle subgroups were used incrementally in the sensitivity test on number of muscles. All other models used a four-muscle input: temporalis-masseter-zygomaticomandibularis-pterygoid.

In order to provide this context with which to evaluate the effect of different modeling parameters on the resulting stresses and strains in comparative mammalian mandible FE models, we conducted sensitivity analyses on a model of the carnivoran *Canis lupus* by testing a range of values for seven required parameters that vary among comparative FE studies (Table 1, Fig. 1). The effects of variation in those parameters on performance measures were evaluated by examining bite force output, strain energy, temporomandibular joint reaction forces, and stress distribution [30]. Bite force output (or other related measures, such as mechanical advantage) is a key performance variable in evaluating and comparing masticatory function of the craniodental system, as larger bite forces permit a species to consume harder and tougher foods, as well as predating on large prey. Both of these adaptations mediate the ecological interactions within the predator guild and across trophic levels. Strain energy has been used as a measure of the work-efficiency of the craniodental system under simulated loads [30]. Selection should favor such work efficiency given the functional demands and trade-offs of achieving maximum stiffness with a given structural quantity and weight (i.e. bone). Joint reaction forces have been shown to exhibit consistent patterns during the mastication cycle, and represent indicators of whether the joint region is being properly modeled [32]. Distributions of von Mises stress is used to show likely areas of failure when the bone undergoes ductile fracture [19], [33]. This study aims to test how input assumptions in FE models affect these performance measures, which are in turn used to test functional hypotheses and in comparisons of functional capability across species.

**Table 1:** Sensitivity tests performed in this study.

Parameter	# Models	Tests
Number of elements	8	Increasing element quantity from 101,674 to 1,404,279
Balancing-Working Ratio	11	+0.1 increment of ratio from 0 to 1.0
Muscle ratio	8	PCSA, mass, dry skull estimates plus individual muscle groups
Muscle number	7	Temporalis only to 6 subgroups of the temporalis and masseter
Bite point constraint	6	Single node constraint to area with 66 nodes
TMJ constraint	4	single node, single link, row of nodes, row of links
Material properties	6	Homogeneous model to 10-property heterogeneous model

A total of 44 models of the same mandible were used in the analyses; some models were used in multiple test categories.

## Materials and Methods

We used the Gray Wolf *Canis lupus* mandible model from Tseng and Binder [26] for sensitivity tests. The structure of interest included both dentaries of the specimen. The specimen was CT-scanned with a Siemens Definition 64 scanner (Siemens Medical Solutions, Forcheim, Germany) with 0.6 mm slice thickness, 0.37 mm pixel resolution, and a size of 512×512 pixels. 499 images were obtained. We chose the mandible for modeling and sensitivity analysis because of the simplicity of the lower jaw, which is composed of two dentary bones with three joints and no sutures [34]. Compared to the cranium, the function of the mandible is not complicated by the multitude of roles, such as the protection of several sensory organs, played by the former structure. In addition, cranial sutures render the cranium a composite structure, and may mediate the location and magnitude of strain during muscle contraction and mastication [35]. Fewer anatomical features need to be accounted for when modeling the mandible, therefore allowing us to focus on the choices in model resolution, material properties, and boundary conditions and their effects on analysis results.

Models were constructed following the protocol used in Dumont et al. [19], [30] and Tseng and Binder [26]. The starting point for the tests was a base model with 383,319 4-noded tetrahedral elements, 0.6 balancing-working side ratio, 55%-26(9)%-10% temporalis-masseter(zygomaticomandibularis)-pterygoid muscle ratio, single-node bite point and TMJ constraints, and a single material property (E = 20 GPa, Poisson ratio = 0.3). All models simulated the biological phenomenon of a unilateral bite with the left lower first molar (the carnassial tooth). We isolated seven main parameters in FE model-building for our sensitivity tests: number of finite elements used to represent the mandibular morphology, balancing- versus working-side muscle activation ratios, relative muscle forces among the masticatory muscle groups, the number of sub-groups within each masticatory muscle group, the size of the bite point constraint, the constraint used at the temporomandibular joints, and number of material properties assigned (Table S1). All models had linear elasticity, and static equilibrium equations were solved in analyses. The variations tested within each category are described in more detail below.

### 1. Number of finite elements

Craniodental FE models in the current literature are mainly built from four-noded tetrahedral elements; these constant stress elements have three degrees of translation freedom per node. Likewise, all models analyzed in this study used four-noded tetrahedrals only. In contrast, ten-noded tetrahedral elements provide more detailed information regarding the distribution of stress and strain within each element, but craniodental FE models built at ∼250,000 elements showed variation in results with 10% between four- versus ten-noded elements [19]. This observation has been cited to justify using four-noded tetrahedral models built with large numbers of elements (>1,000,000) as being sufficient for the general functional questions being asked. Given the current widespread use of four-noded elements in craniodental and in fact most other FE models, we ran eight tests with the more commonly used four-noded tetrahedral elements. Number of triangular elements in each model were adjusted in Geomagic Studio 10 (Geomagic, Inc.) before they were meshed into 4-noded tetrahedral elements in Strand7 2.4.1 (G+D Computer Pty Ltd, Sydney, Australia). The number of tetrahedral elements ranged from ∼100,000 to ∼1,400,000, typical of the resolution seen in most published FE studies (Table S2).

### 2. Muscle activation schemes

Many of the currently published craniodental FE models use symmetrical bilateral muscle forces, even in unilateral biting simulations. Electromyography studies have shown, however, that at least in *Canis*, mastication of bone and meat is achieved without maximum bilateral recruitment of the jaw muscles [36]. Feedback from periodental nerves also plays a role in mediating the use of muscle forces to produce large bite forces, at the same time maintaining joint stability [36]. Therefore, unilateral bite simulations with maximum bilateral muscle recruitment may represent theoretical maxima and not realistic voluntary maxima [37]. Among mammals, a range of muscle recruitment ratios is present both within individuals and across clades; the adaptation of the mandible to particular modes of muscle loading may be informative in themselves in reflecting typical loading scenarios in a given species [36], [38]. We tested unilateral bites at the carnassial tooth (lower molar 1) with 11 models ranging from no balancing side muscle contribution to fully bilateral muscle activation. Working- and balancing-side muscle differences were tested in 10% increments (Table S3). This range encompasses the ratios observed in several mammalian groups [36], [38].

### 3. Muscle proportions

The relative contributions of the three main jaw-closing muscles (temporalis, masseter, and pterygoid) have been estimated in craniodental FE models using either physiological cross-sectional area (PCSA), an estimated of muscle cross-sectional area using dry skulls [39], or by mass of dissected muscles. PCSA has been shown to be a good predictor of muscle force and bite force in bats [28], but in most cases this information is not available for living vertebrates, let along fossil species. We tested a wide range of muscle proportions which encompasses several estimates that have been made for *Canis* [40], [41]. Eight models were made, including muscle activation of each of the three major jaw-closing muscle groups in isolation (represented by numbers in the order of temporalis-masseter-pterygoid; Table S4). Even though the masticatory muscle groups do not activate in isolation in reality, their contribution to, and effects on, the resulting biomechanical performance measures can nevertheless reflect potential adaptations [42]. The lateral pterygoid muscle is proportionally smaller than the other jaw muscles mentioned, accounting for about 3% of total PCSA or <0.6% by wet weight in *Canis lupus* [40]; thus, this relatively minor muscle was not included in our analysis.

### 4. Number of muscles

The main jaw-closing muscles temporalis, masseter, and pterygoid can be subdivided into subgroups based on their gross anatomy, and mammalian craniodental models have been made with a range of muscle groups from temporalis and masseter muscles only [19] to all three muscles and their subgroups [22]. We tested for the effect of number of muscle groups on model outcomes by building seven models ranging from a single jaw closing muscle to seven muscle groups, including subgroups of the temporalis and masseter muscles (Fig. 1, Table S5). The total input force remained the same, and the relative contributions of muscle groups are calculated from the initial 55%-26(9)%-10% temporalis-masseter(zygomaticomandibularis)-pterygoid muscle ratio used in other test categories. Forces among additional subgroups within each major muscle group (when present) are distributed by their respective surface areas.

### 5. Bite point constraint

The evaluation of bite force in craniodental FE models is often done by sampling reaction forces of nodal constraints at the bite points; however, the range of variation in bite force magnitude estimated by a single node constraint versus constraint distributed over an area is unknown. In carnivorans with self-sharpening carnassial teeth, the cusps remain pointed through time, and thus the first point of contact during mastication is situated near the tip of the crown. Therefore, we varied the number of nodes representing the bite point, starting with a single constraint at the tip of the carnassial protoconid. We tested a range of nodal constraint quantity from a single node to ∼65 nodes (covering the entire cusp) using six models (Table S6).

### 6. The temporomandibular joint (TMJ)

The TMJ has been modeled as rotating around single nodes [19], a row of nodes along the condyloid fossa/mandibular condyle [25], or around a beam through the axis of rotation connected to the joints by rigid links [22]. The use of single nodes creates artificially elevated stress values immediately around the constraint, but the overall distribution of stress in the structure is not affected further away from those constraints. We tested four ways of constraining the TMJ in order to examine their differences: (1) single node constraint at each TMJ; (2) a row of nodal constraints along the mandibular condyle; (3) a single rigid link between the mandibular condyle and a beam in the axis of the TMJ with no translation or rotation other than rotation in the dorsoventral plane; (4) rows of rigid links connecting the mandibular fossa to the axial beam with dorsoventral rotational freedom (Table S7). More recently, Dumont et al. [43] used another method to constrain the TMJ, namely allowing translation in the axis connecting the left and right TMJ, in addition to rotational freedom in the sagittal plane. This alternative was not tested in the current study.

### 7. Number of material properties

Two main methods of material property assignments in the current literature on craniodental FEA are (1) assigning properties of a single material to the entire model, or (2) assigning multiple categories of materials based on Hounsfield Units (HU), the gray values representing densities in CT image data. We tested six models ranging from homogeneous single-property to heterogeneous 10-property models (Table S8). Bone properties were assigned based on HU intervals obtained during examination of the CT data using published HU-density and density-modulus equations [44], [45], and tooth enamel and dentine were assigned properties based on published values [46]–[48]. No calibration standard was available from the CT data; the density and modulus equations were applied assuming similar relationships existed for the data (for example, cortical bone properties calculated using unadjusted HU from the CT data provided a density of 1.77 g/cm^3^ and Young's modulus of 19.39 GPa, well within measured range of typical mammalian cortical bone). All materials were treated as isotropic, and all analyses were linear static (Tables S8, S9).

Three-dimensional reconstructions were built from CT image data in Mimics 13.1 (Materialise N.V., Leuven, Belgium), reconstructions were cleaned in Geomagic Studio 10 (Geomagic, Inc., Research Triangle Park, North Carolina, USA), and then remeshed in Mimics. The solid mesh FE models were built in Strand7 2.4.1. The cranium of the specimen was used as reference to identify the direction of muscle forces on the mandible; the relative positions of the cranium and mandible were modified from zero load state (full occlusion, 0° gape) by a 10° rotation of the mandible about the TMJ. This modification created a 10° gape angle that simulated mastication of a small food item between the carnassial teeth. Segmentation of the reconstruction from image data was done using both automated functions in Mimics, as well as manual delineation of bone boundaries. Meshes represented the overall macrostructure of the mandible, without differentiation of microstructural architecture in bone or teeth. Masticatory muscle forces were modeled using the Boneload program written by Grosse et al. [49]. 1000 N of total muscle force was used for all models tested. The model results used for comparison of sensitivity tests were the reaction forces (in Newtons) at the carnassial bite position and the working- and balancing-side joint constraints. Total strain energy (equivalent to the work done in deforming the mandible) values were also compared [30]. In addition, the von Mises stress distributions were visualized on the models. A total of 44 models of the *Canis lupus* mandible were constructed, each given a unique identification number (Table 1, S1; models deposited at Dryad: doi:10.5061/dryad.8961).

## Results

### 1. Number of finite elements

**Figure 2:**
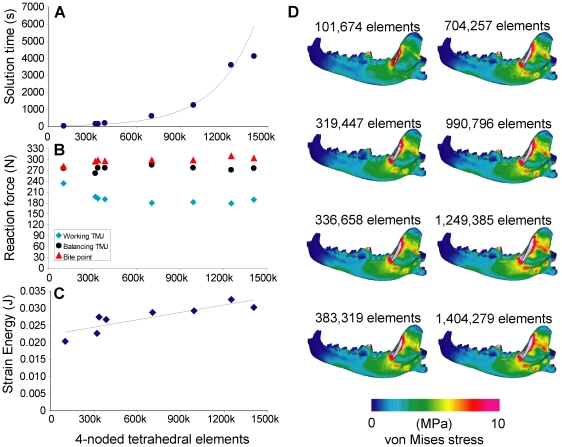
Sensitivity test on tetrahedral element quantity. **A**. Element quantity plotted against solution time (in seconds), with exponential curve in background. **B**. Element quantity plotted against reaction force (in Newtons). **C**. Element quantity plotted against strain energy (in Joules), with linear regression line. **D**. von Mises stress distribution in the working-side dentary in test models; lateral view (in Megapascals).

Reaction forces at the bite point and balancing-side joint were lower in the low resolution model (101,674 elements), and working-side joint forces higher, than all of the other models. Higher-resolution models showed no clear trend in increasing or decreasing reaction forces, although some variation is present (Fig. 2). Strain energy values showed small increase with tetrahedral element quantity, but the slope was on the order of 10^−9^ and does not represent a significant trend. Model solution time increased exponentially between ∼100,000 and ∼1,200,000 tetrahedral elements. The low-resolution model showed lower von Mises stress distributions across the ascending ramus than all other models, which do not show visible differences in stress distribution (Fig. 2D)

### 2. Muscle activation schemes

**Figure 3:**
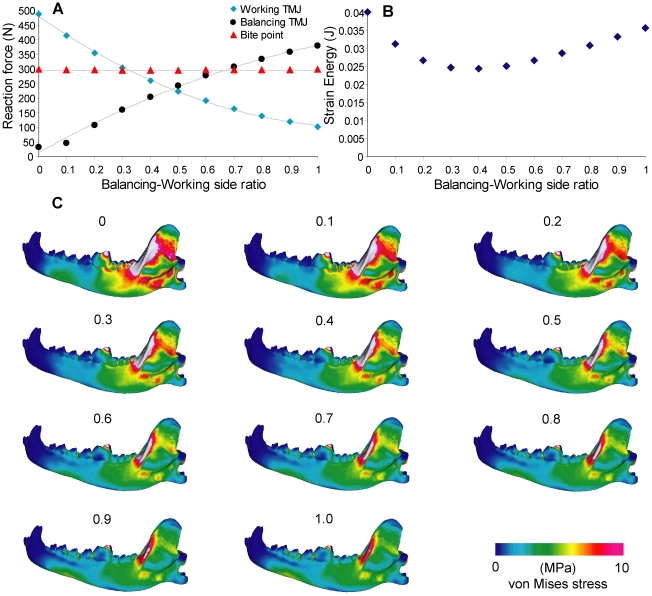
Sensitivity test on balancing-working side ratio. **A**. Ratio plotted against reaction force, with second-order polynomial curves fitted onto the working and balancing reaction forces. **B**. Ratio plotted against strain energy. **C**. von Mises stress distribution in the working-side dentary in test models.

Balancing-side reaction forces increased, and working-side decreased, with increasing ratio of balancing-working side muscle activation (Fig. 3). Bite force remained largely invariant. Joint reaction forces are lower than the bite force on both working- and balancing-sides between the ratios 0.4 to 0.6. Balancing-side joint reaction forces are higher than working-side reaction forces, a pattern consistent with experimental values, at ratios larger than 0.5 [32]. Strain energy values are lowest between ratios of 0.3 to 0.5, and are elevated in both higher and lower ratios. Higher balancing-side muscle activation is correlated with decreased von Mises stress on the working-side ascending ramus, but increased stress in the mandibular corpus below the premolars (Fig. 3C).

### 3. Muscle proportions

**Figure 4:**
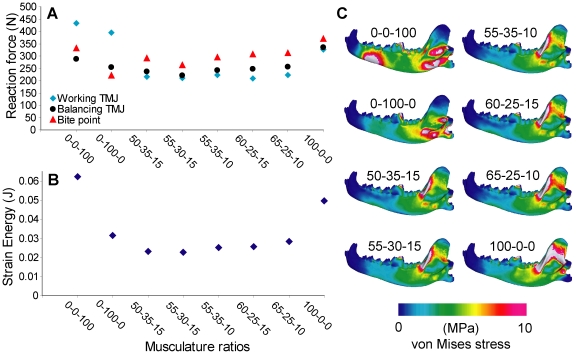
Sensitivity test on musculature ratio. **A**. Ratio plotted against reaction force. **B**. Ratio plotted against strain energy. **C**. von Mises stress distribution in the working-side dentary in test models. Ratios are given by temporalis-masseter-pterygoid sequences, with zygomaticomandibularis considered part of the masseter group.

Using the internal pterygoideus or the masseter muscle in isolation created elevated working-side TMJ reaction forces (Fig. 4). Strain energy values increased when the pterygoideus and temporalis muscles were used in isolation. All other muscle ratios exhibited comparable levels of reaction forces and strain energy values, with the lowest bite force in the 55-30-15 (temporalis-masseter-pterygoideus) model. The ventral side of the mandibular corpus is more stressed in masseter- and pterygoideus-only models, and the ascending ramus is more stressed in temporalis-only models. All other models showed little difference in von Mises stress distribution (Fig. 4C).

### 4. Number of muscles

**Figure 5:**
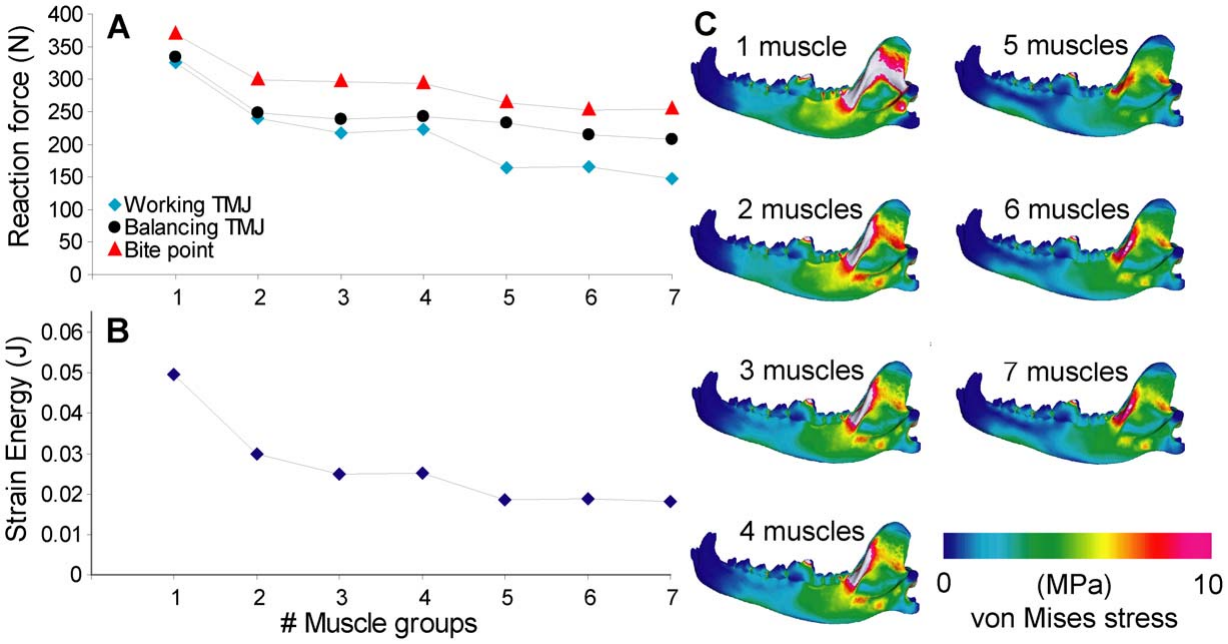
Sensitivity test on number of muscle groups. **A**. Number of groups plotted against reaction force, connected by lines to show trend. **B**. Number of groups plotted against strain energy. **C**. von Mises stress distribution in the working-side dentary in test models.

Reaction forces and strain energy values decreased with increasing number of muscle subgroups modeled. Reaction forces decreased by 20% from the one-muscle model to the 2–4 muscle models, and the latter showed little difference among themselves. A further decrease of ∼15% was observed from the 2–4 muscle models to the 5–7 muscle models; again, the latter group showed little difference among themselves. A larger drop in strain energy (40%) was observed from one-muscle to 2–4 muscle models, and a ∼25% drop from 2–4 muscle models to 5–7 muscle models. Models with more muscle subgroups showed lower stresses in the ascending ramus and the corpus ventral of the premolars (Fig. 5).

### 5. Bite point constraint

**Figure 6:**
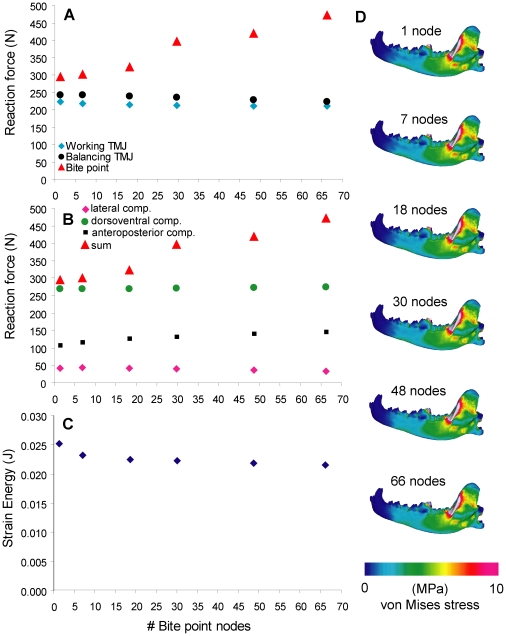
Sensitivity test on nodes at the bite point constraint. **A**. Nodal constraints plotted against reaction force. **B**. Nodal constraints plotted against reaction force, showing components of the bite force vector. **C**. Nodal constraints plotted against strain energy. **D**. von Mises stress distribution in the working-side dentary in test models.

Bite force increased by 60% from a single-node bite point to a 66-node bite point, whereas joint reaction forces stayed constant. Strain energy decreased by <10% from a single-node to a 6-node constraint, but stayed constant for higher numbered constraints. Components of the bite force vector show no significant increases with node number, indicating that the directions of the vector were instead becoming more aligned in the dorsoventral direction, increasing the magnitude of the resultant (Fig. 6B). No differences in stress patterns are observed across the models in areas other than immediately around the bite point, which showed more widespread stress with higher numbered node constraints (Fig. 6).

### 6. The temporomandibular joint (TMJ)

**Figure 7:**
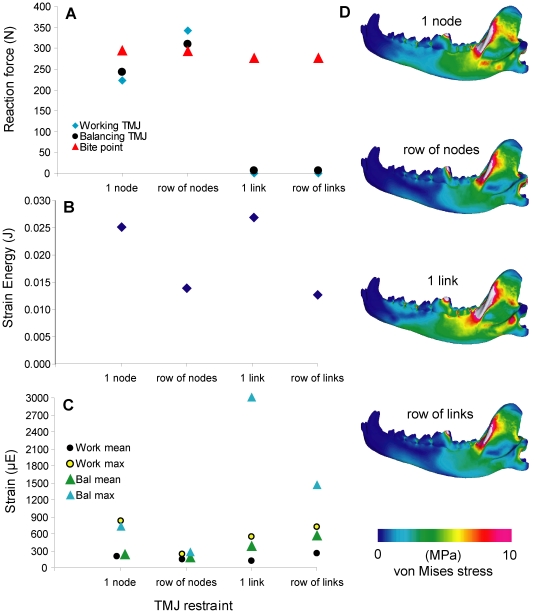
Sensitivity test on temporomandibular joint constraint. **A**. Constraint type plotted against reaction force. **B**. Constraint type plotted against strain energy. **C**. Constraint type plotted against von Mises strain, showing mean and maximum strain for the working- and balancing-side joints, respectively. **D**. von Mises stress distribution in the working-side dentary in test models.

The 10-node model had similar bite force to the 1-node model, but the former had elevated joint reaction forces that exceeded the bite force, and higher working-side TMJ forces than balancing side forces. Bite force decreased <10% in the link models, which had no joint reaction forces at the nodes. Strain energy values decreased by ∼50% from single-node/single-link models to 10-node/10-link models, respectively. Von Mises strain is higher in the link models than in the node models. The single-node/single-link models showed higher von Mises stress in the caudal half of the mandible compared to the other models (Fig. 7).

### 7. Number of material properties

**Figure 8:**
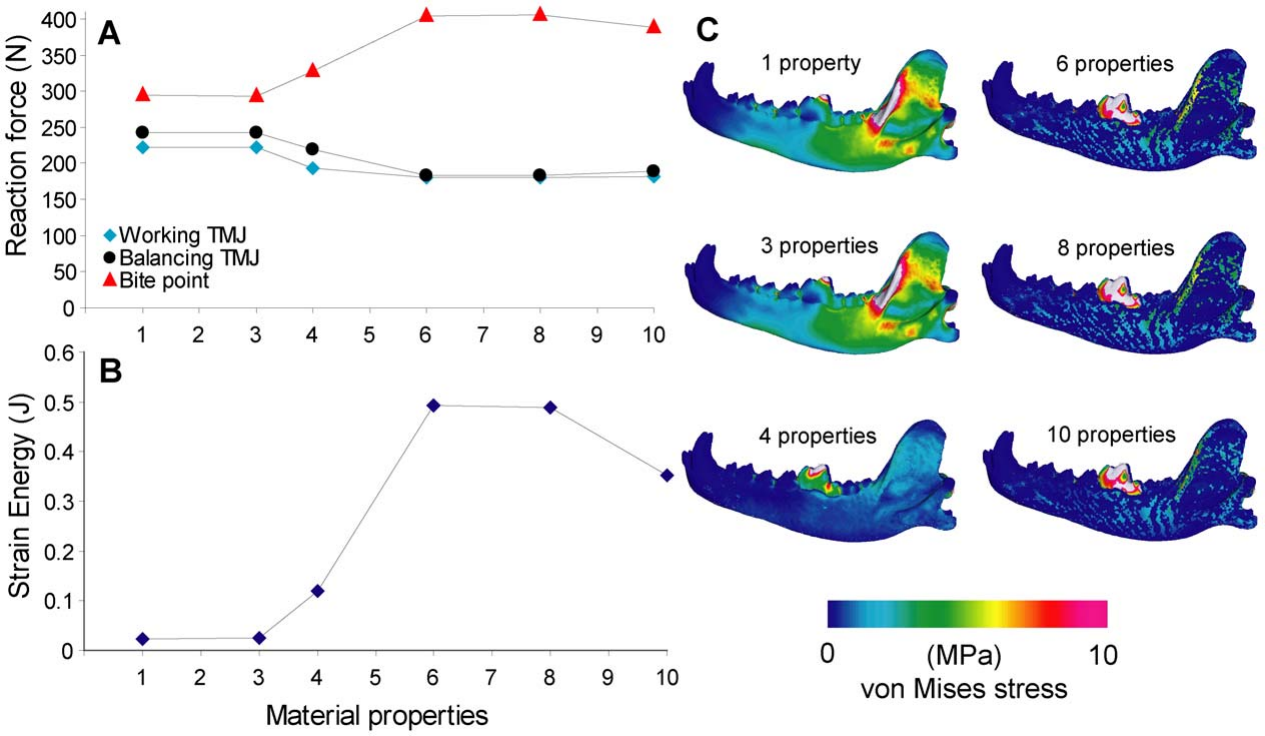
Sensitivity test on number of material properties. **A**. Number of properties plotted against reaction force, connected by lines to show trend. **B**. Number of properties plotted against strain energy. **C**. von Mises stress distribution in the working-side dentary in test models.

Bite force increased by 30%, and joint reaction forces decreased by 20%, from 1–3 property models to 6–8 property models. Strain energy values increased more than 20 fold between those models. The modeling of enamel and dentin had a significant effect on the stress distribution of the models, with most of the stress being contained at the biting tooth in the 6–10 property models (Fig. 8C).

## Discussion

We conducted sensitivity tests on performance measures by altering seven input parameters that are required in FE modeling building, but which vary among comparative studies in the literature. Results showed that varying the values of initial parameters had a wide range of effects on bite force (1% to 60% maximum difference) and strain energy (14.7% to >100% maximum difference). The balancing-working muscle activation ratio had the smallest effect on bite force output over the range tested (0.0–1.0), and for estimates of unilateral bite force one might be tempted to discount its influence on the results. However, plots of changes in joint reaction forces showed that only above a ratio of 0.5 were working-side reaction forces smaller than balancing side reaction forces, as predicted by experimental data (Fig. 3A) [32]. Furthermore, the joint reaction forces were lowest relative to the bite force, and therefore the simulated bite was least stressful to the TMJ, in the 0.4–0.6 ratio range. This range overlaps with the 0.6 ratio obtained experimentally by Dessem [36], who suggested that balancing-side muscle is not fully activated during maximum bite force production, partly because of the need to stabilize the jaw joints. The lowered joint reaction forces observed in the FE models are consistent with this hypothesis. In addition, strain energy values are also lowest in the 0.4–0.5 range, suggesting that this configuration provides optimal mandible performance on the basis of work-efficiency [30]. Even though von Mises stress distributions on the mandible showed no significant differences across the range of ratios tested, using an activation ratio of 0.4 to 0.6 between the balancing- and working-side jaw musculature returned lower joint reaction forces and higher work-efficiency (Fig. 3A) [25], [50].

Bite force output showed most significant changes in models that differed in number of bite point constraints (Fig. 6). Constraints that cover a larger area of contact produced higher bite forces than single-node constraints, and this difference approached 60%. In estimating bite forces, comparative FEA studies have used both a distributed area of bite point constraints [22] and single nodes [28]. Results from our analyses showed that, everything being equal, sum of forces from a multi-node constraint would be larger than in the single-node estimate. In most cases comparative FEA are consistent in their model constraints within each study, but care must be taken when one attempts to evaluate bite forces estimated from different studies with different approaches. This is especially true for extinct taxa; where possible, taxon-specific validation should be coupled with modeling different bite constraints to test the range of reasonable bite force estimates that can be made by FE models [28]. It remains difficult to use FEA for bite force estimates of extinct organisms before generalizations are made on how best to model bite points. Furthermore, the increasing number of constraints placed at the biting tooth could have over-constrained the models beyond realistic scenarios, and this would partially explain the large differences in bite force observed.

Strain energy values were least affected by the type of bite point constraint (Fig. 6C), but were significantly more variable in models that differed in number of material properties (Fig. 8B). This is to be expected because increased number of material properties also created more elements that have lower density and modulus values in the model. Interestingly, very high strain energy (i.e. low work efficiency) was observed in models that had more than six material properties, and von Mises stress in those models are concentrated in the biting tooth (Fig. 8C). The stress distribution indicates that most of the deformation in models with more material properties was concentrated on and within the biting tooth, which was modeled with a plate covering of enamel, and a single-element thick layer of dentine. The large difference that exists in material properties between the tooth and the surrounding bone may explain stress concentration in the former. Evolutionary specializations of enamel microstructure in durophagous carnivorans are consistent with increased selection for stronger teeth, which are required to withstand large stresses incurred from contact against hard food [51], [52]. However, increased stress concentration in the biting tooth was not observed until at least four material properties were present (Fig. 8C), indicating that sufficient differentiation in tooth-bone material properties are required to model this effect. For applications in extinct taxa, fossilized bone often does not provide enough resolution or faithful reproduction of relative bone densities to enable such tests [53]. In cases where such differentiation is possible, however, multiple-property models would tend to increase bite force and also strain energy, and would need to be standardized before comparisons are made across homogeneous and heterogeneous models. The current study did not explicitly consider variation in the ranges of material properties represented in multi-property models. Models with increasing number of material properties also had increasing ranges of densities and modulus values represented by those properties; it remains to be seen how wider or narrower ranges of material properties for a given multi-property model can affect results. An additional factor that has been validated in FE models recently is the localized effect of periodontal ligament on strain in the alveolus; the effect of excluding this tissue from FE models on overall results appear to be small, however [54].

A summary of maximum changes in bite force and strain energy is shown in Table 2. In all but one case, variation in model parameters had larger effects on strain energy than on bite force. In addition, increasing the complexity and magnitude of the values within each parameter can either increase or decrease the performance variables. Theoretically, using a mosaic combination of values in comparisons of any two species models can produce differences where there is none (false positive), or a result of no difference when a difference actually exists (false negative). Functional factors behind a two-model comparison can, therefore, be confounded with variation in input parameters. Whereas balancing-working muscle ratios, bite point and joint constraints, and number of material properties are often standardized across species models, and therefore should not constitute as large a source of error, the number of elements and musculature ratios are rarely identical among currently published models. On average, the doubling of tetrahedral elements in the mandible model led to a ∼12% increase in strain energy. One reason that differences in element numbers change the magnitude of performance measures is the different internal densities of elements as dictated by automated meshing functions in the FE software program. In the program used for this study (Strand7), coarse models are calculated by minimizing steps required to transition to the maximum element size (which is determined by the initial surface mesh), whereas fine models are built without much constraint on numbers of elements with maximum size. As a result, finer models contain larger quantity of small elements. Compounded with the fact that the number of small elements tend to be higher within each model in regions of high curvature or shape change, stress increases can be observed without changing inputs other than element quantity. The number of elements required to consistently represent a model of complex morphology can only be acquired through convergence analyses of each unique model, and a recent study by Bright and Rayfield [55] provides a specific example of convergence analysis in mammalian cranium models.

**Table 2:** Maximum % changes in bite force and strain energy in the sensitivity tests.

Parameter	ΔValue	maxΔ Bite force	maxΔ Strain Energy
Number of elements	102 k–1404 k	+10.2%	+60.0%
Balancing-Working Ratio	0.0–1.0	−1.1%	−39.2%
Muscle ratio	Ptery.-Temp.	+12.0%	−63.7%
Muscle number	1–7	−31.6%	−63.3%
Bite point constraint	1–66	+60.0%	−14.7%
TMJ constraint	nodes-links	−6.3%	−49.6%
Material properties	1–10	+38.2%	+>100%

Value ranges given are for the full range of tests conducted. Changes in bite force and strain energy are maximum differences within the range of each test. **Ptery.**, pterygoid muscles; **temp**., temporalis muscles.

Findings also show that musculature ratios that span the available estimates for *Canis* can produce a ∼20% difference in bite force and 25% difference in strain energy in otherwise identical models. PCSA has been shown to be a good predictor of bite force in bats [28], but in comparisons where PCSA is not available, the results indicate that higher estimates of temporalis would tend to return higher bite force and strain energy values. The pattern of performance changes with changing musculature ratios is inherently interesting, and may reveal functional traits not apparent with comparisons of single models [42]. In these cases building multiple models from the same individual with different musculature ratios would be more informative than choosing among the available means of estimates of masticatory muscle force to build a single model.

In summary, the variations that arise in FEA results from changing initial parameters can be confounded with functional differences in model comparisons. More confidence can be placed in model comparisons where these factors are examined by sensitivity and convergence analyses, and in some cases standardized. In standardizing models, it is more important to keep bite point constraints and the number and range of material properties constant in evaluating bite force outputs, and keeping material properties, musculature ratios, and muscle subgroups constant for strain energy comparisons. The relationship between TMJ joint reaction forces on the balancing- versus working side jaw should be examined along with bite forces to ensure the forces acting on the models are reasonably comparable to experimental results. Visual stress distributions are affected more by number of material properties than by any other factor examined. Comparisons between different modeling protocols, if they are to be made, should consider these influences.

### Other parameters

The cross-sectional shape of mandibles is an important predictor of feeding performance and bending strength in carnivorans [56]. However, in studies of fossil species the internal structures of the skull may not be preserved, and in some cases filled models can provide reasonable estimates of mechanical behavior in the original morphology [26]. In cases where internal morphology simply cannot be reconstructed with any confidence, the filled models may be sufficient for broad comparison purposes. However, researchers may wish to reconstruct the internal cavity by approximating its boundary if the evolution of corpus cortical thickness is of interest.

The mandibular symphysis, which exhibits variation in composition and gross anatomy across mammal species, is a key location that affects the distribution of stresses across the dentaries [34]. Tseng and Stynder [57] tested a range of material properties to approximate the mechanical behavior of the mandibular symphysis in their carnivoran models, and found that in most cases the stress is conducted through the symphysis, but modeling the joint as cortical bone can increase regional stress. Their results are superficially similar to those presented by McHenry et al. [22] and Wroe et al. [21], and suggest that at least for the symphysis, those models show elevated stress compared to ones constructed with material properties closer to ligament or fibrocartilage.

Homogeneous models, which are built using a single set of material properties, usually representing average cortical bone, are common in comparative studies [21], [23], [25]. A sensitivity test of typical elastic modulus and Poisson ratio values used in construction of homogeneous models was conduced by Tseng et al. [53], who showed that the middle range of elastic modulus (15–30 GPa) and Poisson ratio (0.1–0.4) used by many studies gave comparable results in stress and strain. Thus, stress distributions of homogeneous models built with values within those ranges are not expected to be significantly influenced by modeling artifacts when used in comparisons.

The sensitivity tests performed in this study are by no means exhaustive, and the range of input assumptions represented by the current set of models can be expanded upon to include more extensive or specific tests that pertain to specific research questions. The models discussed in this study are available in the Dryad Digital Repository (doi:10.5061/dryad.8961).

### Conclusions

We conducted a series of sensitivity tests to evaluate the range of variation among the modeling parameters required in studies of functional morphology using FEA. Findings indicate that not all parameters are equally variable, and consideration needs to be given to particular sets of parameters, based on the research question being asked. In a purely comparative context, a Gray Wolf mandible model required only ∼300,000 elements to produce reaction forces and strain energy values close to those obtained from higher-resolution models (>1,400,000 elements). Whereas PCSA, mass, or other estimates of muscle ratios did not greatly affect the results, the adjustment of the balancing-working side ratio in unilateral biting simulation does have an effect on joint reaction forces. For comparative purposes, the number of muscle subgroups, the area of bite point constraints, the TMJ constraint, and the number and range of material properties should be kept consistent across models within a single study. Across different studies, the compound effects of variation among those factors may be large, and differences up to 50% can be observed by extreme values in a single parameter. Validation of FE models of living species is needed to determine the set of input parameters that would give the most realistic results in a given study, but comparative studies can nevertheless be highly informative especially if sources of variation can be identified within the particular set of values used to construct the models. Lastly, the pattern of variation obtained through tests of a given parameter within each model may be instructive in itself, thus researchers may wish to consider sensitivity tests as part of a study design of comparative form and function using FEA.

## Supporting Information

Table S1 **Models used in the sensitivity tests.** Models and their descriptions are available at Dryad Digital Repository: doi:10.5061/dryad.8961.
(PDF)

Table S2 **Data for sensitivity test 1: Number of tetrahedral elements.** **Tet4**, number of 4-noded tetrahedral elements; **SE**, strain energy.
(PDF)

Table S3 **Data for sensitivity test 2: balancing-working muscle ratios.**
(PDF)

Table S4 **Data for sensitivity test 3: musculature ratios.**
(PDF)

Table S5 **Data for sensitivity test 4: number of muscle subgroups.**
(PDF)

Table S6 **Data for sensitivity test 5: bite point constraint.** **m1.ap**, anteroposterior component, **m1.dv**, dorsoventral component, **m1.lat**, lateral component.
(PDF)

Table S7 **Data for sensitivity test 6: temporomandibular joint constraints.**
(PDF)

Table S8 **Data for sensitivity test 7: number of material properties.**
(PDF)

Table S9 **Material properties used in sensitivity test 7.** **HU**, Hounsfield Units; **E**, elastic (Young's) modulus; **ν**, Poisson ratio. Material properties of bone were derived from the regression equation of Schneider et al. (1996): Density (g/cm^3^) = 0.0007*HU+0.3489, R^2^ = 0.9994, and modulus values were calculated from the regression equation of Rho et al. (1995) as E (GPa) = 5.05*ρ^1.269^ for ρ<1 g/cm3 and E (GPa) = 9.11*ρ^1.326^ for ρ>1 g/cm^3^. The lowest density material was arbitrarily assigned ρ = 0.01 g/cm^3^ as a low-end value.
(PDF)

## References

[1] Rayfield EJ (2007). Finite element analysis and understanding the biomechanics and evolution of living and fossil organisms.. Annual Review of Earth and Planetary Science.

[2] Ross CF (2005). Finite element analysis in vertebrate biomechanics.. The Anatomical Record.

[3] Kupczik K (2008). Virtual biomechanics: basic concepts and technical aspects of finite element analysis in vertebrate morphology.. Journal of Anthropological Sciences.

[4] Fastnacht M, Hess N, Frey E, Weiser H-P (2002). Finite element analysis in vertebrate palaeontology.. Senckenbergiana lethaea.

[5] McHenry C, Clausen PD, Daniel WJT, Meers MB, Pendharkar A (2006). Biomechanics of the rostrum in crocodilians, a comparative analysis using finite element modeling.. The Anatomical Record.

[6] Bell PE, Snively E, Shychoski L (2009). A comparison of the jaw mechanics in hadrosaurid and ceratopsid dinosaurs using finite element analysis.. The Anatomical Record.

[7] Xing L, Ye Y, Shu C, Peng G, You H (2009). Structure, orientation and finite element analysis of the tail club of *Mamenchisaurus hochuanensis*.. Acta Geologica Sinica.

[8] Arbour VM, Snively E (2009). Finite element analyses of ankylosaurid dinosaur tail club impacts.. The Anatomical Record.

[9] Rayfield EJ, Norman DB, Jorner CC, Horner JR, Smith PM (2001). Cranial design and function in a large theropod dinosaur.. Nature.

[10] Rayfield EJ (2004). Cranial mechanics and feeding in *Tyrannosaurus rex*.. Proceedings of the Royal Society of London, Series B.

[11] Rayfield EJ (2005). Aspects of comparative cranial mechanics in the theropod dinosaurs *Coelophysis*, *Allosaurus*, and *Tyrannosaurus*.. Zoological Journal of the Linnean Society.

[12] Soons J, Herrel A, Genbrugge A, Aerts P, Podos J (2010). Mechanical stress, fracture risk and beak evolution in Darwin's ground finches (*Geospiza*).. Philosophical Transactions of the Royal Society of London B Biological Sciences.

[13] Moazen M, Curtis N, Evans SE, O'Higgins P, Fagan MJ (2008). Combined finite element and multibody dynamics analysis of biting in a *Uromastyx hardwickii* lizard skull.. Journal of Anatomy.

[14] Moreno K, Wroe S, Clausen PD, McHenry C, D'Amore DC (2008). Cranial performance in the Komodo dragon (*Varanus komodoensis*) as revealed by high-resolution 3-D finite element analysis.. Journal of Anatomy.

[15] Hulsey CD, Roberts RJ, Lin ASP, Guldberg R, Streelman JT (2008). Convergence in a mechanically complex phenotype: Detecting structural adaptations for crushing in cichlid fish.. Evolution.

[16] Tanner JB, Dumont ER, Sakai ST, Lundrigan BL, Holekamp KE (2008). Of arcs and vaults: the biomechanics of bone-cracking in spotted hyenas (*Crocuta crocuta*).. Biological Journal of the Linnean Society.

[17] Strait DS, Weber GW, Neubauer S, Chalk J, Richmond BG (2009). The feeding biomechanics and dietary ecology of *Australopithecus africanus*.. Proceedings of the National Academy of Sciences.

[18] Slater GJ, Van Valkenburgh B (2009). Allometry and performance: the evolution of skull form and function in felids.. Journal of Evolutionary Biology.

[19] Dumont ER, Piccirillo J, Grosse IR (2005). Finite-element analysis of biting behavior and bone stress in the facial skeletons of bats.. The Anatomical Record.

[20] Wroe S (2008). Cranial mechanics compared in extinct marsupial and extant African lions using a finite-element approach.. Journal of Zoology.

[21] Wroe S, Clausen PD, McHenry C, Moreno K, Cunningham E (2007). Computer simulation of feeding behaviour in the thylacine and dingo as a novel test for convergence and niche overlap.. Proceedings of the Royal Society B: Biological Sciences.

[22] McHenry C, Wroe S, Clausen PD, Moreno K, Cunningham E (2007). Supermodeled sabercat, predatory behavior in *Smilodon fatalis* revealed by high-resolution 3D computer simulation.. Proceedings of the National Academy of Sciences.

[23] Slater GJ, Dumont ER, Van Valkenburgh B (2009). Implications of predatory specialization for cranial form and function in canids.. Journal of Zoology.

[24] Farke AA (2008). Frontal sinuses and head-butting in goats: a finite element analysis.. The Journal of Experimental Biology.

[25] Tseng ZJ (2009). Cranial function in a late Miocene *Dinocrocuta gigantea* (Mammalia: Carnivora) revealed by comparative finite element analysis.. Biological Journal of the Linnean Society.

[26] Tseng ZJ, Binder WJ (2010). Mandibular biomechanics of *Crocuta crocuta*, *Canis lupus*, and the late Miocene *Dinocrocuta gigantea* (Carnivora, Mammalia).. Zoological Journal of Linnean Society.

[27] Ross CF, Patel BA, Slice DE, Strait DS, Dechow PC (2005). Modeling masticatory muscle force in finite element analysis: sensitivity analysis using principal coordinates analysis.. The Anatomical Record.

[28] Davis JL, Santana SE, Dumont ER, Grosse I (2010). Predicting bite force in mammals: two-dimensional *versus* three-dimensional lever models.. Journal of Experimental Biology.

[29] Strait DS, Wang Q, Dechow PC, Ross CF, Richmond BG (2005). Modeling elastic properties in finite-element analysis: how much precision is needed to produce an accurate model?. The Anatomical Record.

[30] Dumont ER, Grosse I, Slater GJ (2009). Requirements for comparing the performance of finite element models of biological structures.. Journal of Theoretical Biology.

[31] Sellers WI, Crompton RH (2004). Using sensitivity analysis to validate the predictions of a biomechanical model of bite forces.. Annals of Anatomy.

[32] Hylander WL (1979). An experimental analysis of temporomandibular joint reaction force in macaques.. American Journal of Physical Anthropology.

[33] Nalla RK, Kinney JH, Ritchie RO (2003). Mechanistic failure criteria for the failure of human cortical bone.. Nature Materials.

[34] Scapino RP (1965). The third joint of the canine jaw.. Journal of Morphology.

[35] Herring SW, Teng S (2000). Strain in the braincase and its suture during function.. American Journal of Physical Anthropology.

[36] Dessem D (1989). Interactions between jaw-muscle recruitment and jaw-joint forces in *Canis familiaris*.. Journal of Anatomy.

[37] Ellis JL, Thomason JJ, Kebreab E, France J (2008). Calibration of estimated biting forces in domestic canids: comparison of post-mortem and *in vivo* measurements.. Journal of Anatomy.

[38] Hylander WL, Ravosa MJ, Ross CF, Wall CE, Johnson KR (2000). Symphyseal fusion and jaw-adductor muscle force: An EMG study.. American Journal of Physical Anthropology.

[39] Thomason JJ (1991). Cranial strength in relation to estimate biting forces in some mammals.. Canadian Journal of Zoology.

[40] Schumacher GH (1961). Funktionelle Morphologie der Kaumuskulatur.

[41] Turnbull WD (1970). Mammalian masticatory apparatus.. Fieldiana: Geology.

[42] Tseng ZJ, Wang X (2010). Cranial functional morphology of fossil dogs and adaptation for durophagy in *Borophagus* and *Epicyon* (Carnivora, Mammalia).. Journal of Morphology.

[43] Dumont ER, Davis JL, Grosse I, Burrows AM (2011). Finite element analysis of performance in the skulls of marmosets and tamarins.. Journal of Anatomy.

[44] Rho JY, Hobatho MC, Ashman RB (1995). Relations of mechanical properties to density and CT numbers in human bone.. Medical Engineering and Physics.

[45] Schneider U, Pedroni E, Lomax A (1996). The calibration of CT Hounsfield units for radiotherapy treatment planning.. Physics in Medicine and Biology.

[46] Qin Q-H, Swain MV (2004). A micro-mechanics model of dentin mechanical properties.. Biomaterials.

[47] Habelitz S, Marshall SJ, Marshall GW, Balooch M (2001). Mechanical properties of human dental enamel on the nanometre scale.. Archives of Oral Biology.

[48] Haines DJ (1968). Physical properties of human tooth enamel and enamel sheath material under load.. Journal of Biomechanics.

[49] Grosse I, Dumont ER, Coletta C, Tolleson A (2007). Techniques for modeling muscle-induced forces in finite element models of skeletal structures.. The Anatomical Record.

[50] Slater GJ, Figueirido B, Louis L, Yang P, Van Valkenburgh B (2010). Biomechanical consequences of rapid evolution in the polar bear lineage.. PLoS ONE.

[51] Stefen C, Koenigswald Wv, Sander PM (1997). Chapter 7. Differentiations in Hunter-Schreger bands of carnivores.. Tooth enamel microstructure.

[52] Rensberger JM, Thomason JJ (1995). Determination of stresses in mammalian dental enamel and their relevance to the interpretation of feeding behaviors in extinct taxa.. Functional morphology in vertebrate paleontology.

[53] Tseng ZJ, Antón M, Salesa MJ (2011). The evolution of the bone-cracking model in carnivorans: Cranial functional morphology of the Plio-Pleistocene cursorial hyaenid *Chasmaporthetes lunensis* (Mammalia: Carnivora).. Paleobiology.

[54] Panagiotopoulou O, Kupczik K, Cobb SN (2011). The mechanical function of the periodontal ligament in the macaque mandible: a validation and sensitivity study using finite element analysis.. Journal of Anatomy.

[55] Bright JA, Rayfield EJ (2011). The response of cranial biomechanical finite element models to variations in mesh density.. The Anatomical Record.

[56] Biknevicius AR, Ruff CB (1992). The structure of the mandibular corpus and its relationship to feeding behaviors in extant carnivorans.. Journal of Zoology.

[57] Tseng ZJ, Stynder D (2011). Mosaic functionality in a transitional ecomorphology: skull biomechanics in stem Hyaeninae compared to modern South African carnivorans.. Biological Journal of the Linnean Society.

