# Supplementary material for: Model Sensitivity and Use of the Comparative Finite Element Method in Mammalian Jaw Mechanics: Mandible Performance in the Gray Wolf
Source: PLoS One. 2011 Apr 29;6(4):e19171. doi: 10.1371/journal.pone.0019171 (PMC3084775; doi:10.1371/journal.pone.0019171)
Supplement: Table S1 — Models used in the sensitivity tests. Models and their descriptions are available at Dryad Digital Repository: doi:10.5061/dryad.8961. (PDF) [file pone.0019171.s001.pdf]

Table S1. Models used in the sensitivity tests. Models and their descriptions are available at Dryad Digital Repository: doi:10.5061/dryad.8961

Test 1 models	Test 2 models	Test 3 models	Test 4 models	Test 5 models	Test 6 models	Test 7 models
J20101210TSA08	J20101213TSA18	J20101214TSA25	J20101214TSA23	J20101213TSA13	J20101213TSA13	J20101215TSA39
J20101112TSA01	J20101213TSA17	J20101214TSA24	J20101214TSA26	J20101215TSA31	J20101215TSA36	J20101215TSA40
J20101208TSA03	J20101213TSA16	J20101213TSA19	J20101214TSA27	J20101215TSA32	J20101215TSA37	J20101215TSA41
J20101209TSA04	J20101213TSA15	J20101213TSA20	J20101213TSA13	J20101215TSA33	J20101215TSA38	J20101215TSA42
J20101209TSA05	J20101213TSA14	J20101213TSA13	J20101214TSA28	J20101215TSA34		J20101215TSA43
J20101209TSA07	J20101213TSA13	J20101213TSA21	J20101214TSA29	J20101215TSA35		J20101215TSA44
J20101208TSA02	J20101209TSA04	J20101214TSA22	J20101214TSA30			
J20101209TSA06	J20101213TSA12	J20101214TSA23				
	J20101213TSA11					
	J20101213TSA10					
	J20101213TSA09					
