# Supplementary material for: Model Sensitivity and Use of the Comparative Finite Element Method in Mammalian Jaw Mechanics: Mandible Performance in the Gray Wolf
Source: PLoS One. 2011 Apr 29;6(4):e19171. doi: 10.1371/journal.pone.0019171 (PMC3084775; doi:10.1371/journal.pone.0019171)
Supplement: Table S2 — Data for sensitivity test 1: Number of tetrahedral elements. Tet4, number of 4-noded tetrahedral elements; SE, strain energy. (PDF) [file pone.0019171.s002.pdf]

Table S2. Data for sensitivity test 1: Number of tetrahedral elements. **Tet4**, number of 4-noded tetrahedral elements; **SE**, strain energy.

Model	Tet4	SE (J)	m1 (N)	workTMJ (N)	balTMJ (N)	Solution time (s)
J20101210TSA08	101674	0.0203	278.97	234.67	275.72	20
J20101112TSA01	319447	0.0226	292.90	197.11	261.68	152
J20101208TSA03	336658	0.0274	296.26	192.59	276.81	150
J20101209TSA04	383319	0.0267	294.11	190.64	276.59	192
J20101209TSA05	704257	0.0288	296.30	180.04	284.73	609
J20101209TSA07	990796	0.0293	296.36	182.57	277.50	1242
J20101208TSA02	1249385	0.0325	307.58	179.02	271.64	3591
J20101209TSA06	1404279	0.0302	302.02	188.67	275.44	4114
