# Supplementary material for: Model Sensitivity and Use of the Comparative Finite Element Method in Mammalian Jaw Mechanics: Mandible Performance in the Gray Wolf
Source: PLoS One. 2011 Apr 29;6(4):e19171. doi: 10.1371/journal.pone.0019171 (PMC3084775; doi:10.1371/journal.pone.0019171)
Supplement: Table S3 — Data for sensitivity test 2: balancing-working muscle ratios. (PDF) [file pone.0019171.s003.pdf]

Table S3. Data for sensitivity test 2: balancing-working muscle ratios.

Model	Balancing%	SE (J)	m1 (N)	workTMJ (N)	balTMJ (N)
J20101213TSA18	0	0.0401	296.74	486.91	30.75
J20101213TSA17	0.1	0.0313	294.97	414.11	45.07
J20101213TSA16	0.2	0.0267	294.04	353.69	106.58
J20101213TSA15	0.3	0.0247	293.65	302.87	158.85
J20101213TSA14	0.4	0.0244	293.61	259.64	203.69
J20101213TSA13	0.5	0.0252	293.79	222.58	242.57
J20101209TSA04	0.6	0.0267	294.11	190.64	276.59
J20101213TSA12	0.7	0.0287	294.52	163.07	306.61
J20101213TSA11	0.8	0.0309	294.98	139.32	333.29
J20101213TSA10	0.9	0.0333	295.48	119.04	357.18
J20101213TSA09	1	0.0357	295.99	102.06	378.66
