# Supplementary material for: Model Sensitivity and Use of the Comparative Finite Element Method in Mammalian Jaw Mechanics: Mandible Performance in the Gray Wolf
Source: PLoS One. 2011 Apr 29;6(4):e19171. doi: 10.1371/journal.pone.0019171 (PMC3084775; doi:10.1371/journal.pone.0019171)
Supplement: Table S4 — Data for sensitivity test 3: musculature ratios. (PDF) [file pone.0019171.s004.pdf]

Table S4. Data for sensitivity test 3: musculature ratios.

Model	Musculature	SE (J)	m1 (N)	workTMJ (N)	balTMJ (N)
J20101214TSA25	0-0-100	0.0622	329.29	433.45	287.53
J20101214TSA24	0-100-0	0.0314	218.84	394.82	255.06
J20101213TSA19	50-35-15	0.023	289.81	216.55	236.55
J20101213TSA20	55-30-15	0.0226	261.47	210.22	221.82
J20101213TSA13	55-35-10	0.0252	293.79	222.58	242.57
J20101213TSA21	60-25-15	0.0255	305.34	208.90	247.46
J20101214TSA22	65-25-10	0.0282	310.10	222.08	256.10
J20101214TSA23	100-0-0	0.0496	368.57	325.61	334.49
