# Supplementary material for: Model Sensitivity and Use of the Comparative Finite Element Method in Mammalian Jaw Mechanics: Mandible Performance in the Gray Wolf
Source: PLoS One. 2011 Apr 29;6(4):e19171. doi: 10.1371/journal.pone.0019171 (PMC3084775; doi:10.1371/journal.pone.0019171)
Supplement: Table S5 — Data for sensitivity test 4: number of muscle subgroups. (PDF) [file pone.0019171.s005.pdf]

Table S5. Data for sensitivity test 4: number of muscle subgroups.

Model	Muscle groups	SE (J)	m1 (N)	workTMJ (N)	balTMJ (N)
J20101214TSA23	1	0.0496	368.57	325.61	334.49
J20101214TSA26	2	0.0298	298.71	240.68	248.46
J20101214TSA27	3	0.025	295.65	217.98	238.84
J20101213TSA13	4	0.0252	293.79	222.58	242.57
J20101214TSA30	5	0.0185	263.18	164.83	232.79
J20101214TSA29	6	0.0188	252.06	165.40	214.86
J20101214TSA28	7	0.0182	253.83	147.38	207.96

1 muscle: temporalis only
2 muscles: temporalis and masseter (no subgroups)
3 muscles: temporalis, masseter, and internal pterygoid
4 muscles: temporalis, masseter, zygomaticomandibularis, internal pterygoid
5 muscles: temporalis (superficial + deep), zygomatic part of temporalis, masseter, zygomaticomandibularis, internal pterygoid
6 muscles: superficial temporalis, deep temporalis, zygomatic temporalis, masseter, zygomaticomandibularis, internal pterygoid
7 muscles: superficial temporalis, deep temporalis, zygomatic temporalis, superficial masseter, deep masseter, zygomaticomandibularis, internal pterygoid
