# Supplementary material for: Model Sensitivity and Use of the Comparative Finite Element Method in Mammalian Jaw Mechanics: Mandible Performance in the Gray Wolf
Source: PLoS One. 2011 Apr 29;6(4):e19171. doi: 10.1371/journal.pone.0019171 (PMC3084775; doi:10.1371/journal.pone.0019171)
Supplement: Table S6 — Data for sensitivity test 5: bite point constraint. m1.ap, anteroposterior component, m1.dv, dorsoventral component, m1.lat, lateral component. (PDF) [file pone.0019171.s006.pdf]

Table S6. Data for sensitivity test 5: bite point constraint. m1.ap, anteroposterior component, **m1.dv**, dorsoventral component, **m1.lat**, lateral component.

Model	Bite point nodes	SE (J)	workTMJ (N)	balTMJ (N)	m1 (N)	m1.ap (N)	m1.dv (N)	m1.lat (N)
J20101213TSA13	1	0.0252	222.58	242.57	293.79	42.81	269.46	108.95
J20101215TSA31	7	0.0232	218.19	241.64	299.48	43.15	268.50	117.80
J20101215TSA32	18	0.0225	214.80	237.85	321.37	41.37	269.18	127.13
J20101215TSA33	30	0.0223	212.11	235.68	394.43	40.03	270.25	132.93
J20101215TSA34	48	0.0218	210.51	228.69	417.16	36.15	272.80	141.73
J20101215TSA35	66	0.0215	211.00	223.13	470.11	33.08	274.44	146.51
