# Supplementary material for: Model Sensitivity and Use of the Comparative Finite Element Method in Mammalian Jaw Mechanics: Mandible Performance in the Gray Wolf
Source: PLoS One. 2011 Apr 29;6(4):e19171. doi: 10.1371/journal.pone.0019171 (PMC3084775; doi:10.1371/journal.pone.0019171)
Supplement: Table S7 — Data for sensitivity test 6: temporomandibular joint constraints. (PDF) [file pone.0019171.s007.pdf]

Table S7. Data for sensitivity test 6: temporomandibular joint constraints

Model	TMJ	SE (J)	workTMJ (N)	BalTMJ (N)	m1 (N)	workStrain (µE)	workStrain max (µE)	balStrain (µE)	balStrain max (µE)
J20101213TSA13	1 node	0.0252	222.58	242.57	293.79	198	821	221	722
J20101215TSA37	10 nodes	0.0139	341.38	308.95	291.80	148	244	168	261
J20101215TSA36	1 link	0.0269	0.00	0.00	275.95	122	550	371	3000
J20101215TSA38	10 links	0.0127	0.00	0.00	275.40	255	725	555	1450
