# Supplementary material for: Model Sensitivity and Use of the Comparative Finite Element Method in Mammalian Jaw Mechanics: Mandible Performance in the Gray Wolf
Source: PLoS One. 2011 Apr 29;6(4):e19171. doi: 10.1371/journal.pone.0019171 (PMC3084775; doi:10.1371/journal.pone.0019171)
Supplement: Table S8 — Data for sensitivity test 7: number of material properties. (PDF) [file pone.0019171.s008.pdf]

Table S8. Data for sensitivity test 7: number of material properties

Model	Properties	SE (J)	m1 (N)	workTMJ (N)	balTMJ (N)
J20101215TSA39	1	0.0241	293.79	222.58	242.57
J20101215TSA40	3	0.0242	293.71	222.76	242.55
J20101215TSA41	4	0.1192	327.72	192.76	219.20
J20101215TSA42	6	0.4922	403.97	180.69	183.75
J20101215TSA44	8	0.488	405.83	180.65	182.98
J20101215TSA43	10	0.3527	388.16	181.44	189.57
