# Supplementary material for: Model Sensitivity and Use of the Comparative Finite Element Method in Mammalian Jaw Mechanics: Mandible Performance in the Gray Wolf
Source: PLoS One. 2011 Apr 29;6(4):e19171. doi: 10.1371/journal.pone.0019171 (PMC3084775; doi:10.1371/journal.pone.0019171)
Supplement: Table S9 — Material properties used in sensitivity test 7. HU, Hounsfield Units; E, elastic (Young's) modulus; ν, Poisson ratio. Material properties of bone were derived from the regression equation of Schneider et al. (1996): Density (g/cm3) = 0.0007*HU+0.3489, R2 = 0.9994, and modulus values were calculated from the regression equation of Rho et al. (1995) as E (GPa) = 5.05*ρ1.269 for ρ<1 g/cm3 and E (GPa) = 9.11*ρ1.326 for ρ>1 g/cm3. The lowest density material was arbitrarily assigned ρ = 0.01 g/cm3 as a low-end value. (PDF) [file pone.0019171.s009.pdf]

Table S9. Material properties used in sensitivity test 7. HU, Hounsfield Units; **E**, elastic (Young's) modulus; **ν**, Poisson ratio. Material properties of bone were derived from the regression equation of Schneider et al. (1996): Density (g/cm^3) = 0.0007*HU + 0.3489, R^2 = 0.9994, and modulus values were calculated from the regression equation of Rho et al. (1995) as E (GPa) = $5.05*\rho^{1.269}$ for ρ < 1 g/cm3 and E (GPa) = $9.11*\rho^{1.326}$ for ρ > 1 g/cm^3 . The lowest density material was arbitrarily assigned ρ=0.01 g/cm^3 as a low-end value.

	10 properties		8 properties		6 properties		4 properties		3 properties		1 property	
Mean HU	Density (g/cm3)	E (GPa)	Density (g/cm3)	E (GPa)	Density (g/cm3)	E (GPa)	Density (g/cm3)	E (GPa)	v	E (GPa)	v	E (GPa)
-820.75	0.01	0.01	0.03	0.08	0.03	0.08	0.26	1.07	0.30	20.00	0.30	20.00
-413.75	0.06	0.14	0.03	0.08	0.03	0.08	0.26	1.07	0.30	20.00	0.30	20.00
-6.75	0.34	1.30	0.49	2.05	0.49	2.05	0.26	1.07	0.30	20.00	0.30	20.00
400.25	0.63	2.80	0.49	2.05	0.49	2.05	0.26	1.07	0.30	20.00	0.30	20.00
807.25	0.91	4.51	0.91	4.51	1.06	8.05	1.34	12.71	0.30	20.00	0.30	20.00
1214.25	1.20	11.59	1.20	11.59	1.06	8.05	1.34	12.71	0.30	20.00	0.30	20.00
1621.25	1.48	15.37	1.48	15.37	1.63	17.38	1.34	12.71	0.30	20.00	0.30	20.00
2026.5	1.77	19.39	1.77	19.39	1.63	17.38	1.34	12.71	0.30	20.00	0.30	20.00
Dentine	(v = 0.18)	18.80	(v = 0.18)	18.80	(v = 0.18)	18.80	(v = 0.18)	18.80	0.18	18.80	0.30	20.00
Enamel	(v = 0.30)	80.10	(v = 0.30)	80.10	(v = 0.30)	80.10	(v = 0.30)	80.10	0.30	80.10	0.30	20.00
